# Can Survival Prediction Be Improved By Merging Gene Expression Data Sets?

**DOI:** 10.1371/journal.pone.0007431

**Published:** 2009-10-23

**Authors:** Haleh Yasrebi, Peter Sperisen, Viviane Praz, Philipp Bucher

**Affiliations:** 1 Swiss Institute for Experimental Cancer Research (ISREC), Swiss Federal Institute of Technology (EPFL), School of Life Sciences, EPFL SV ISREC, Lausanne, Switzerland; 2 Swiss Institute of Bioinformatics, EPFL SV ISREC, Lausanne, Switzerland; Deutsches Krebsforschungszentrum, Germany

## Abstract

**Background:**

High-throughput gene expression profiling technologies generating a wealth of data, are increasingly used for characterization of tumor biopsies for clinical trials. By applying machine learning algorithms to such clinically documented data sets, one hopes to improve tumor diagnosis, prognosis, as well as prediction of treatment response. However, the limited number of patients enrolled in a single trial study limits the power of machine learning approaches due to over-fitting. One could partially overcome this limitation by merging data from different studies. Nevertheless, such data sets differ from each other with regard to technical biases, patient selection criteria and follow-up treatment. It is therefore not clear at all whether the advantage of increased sample size outweighs the disadvantage of higher heterogeneity of merged data sets. Here, we present a systematic study to answer this question specifically for breast cancer data sets. We use survival prediction based on Cox regression as an assay to measure the added value of merged data sets.

**Results:**

Using time-dependent Receiver Operating Characteristic-Area Under the Curve (ROC-AUC) and hazard ratio as performance measures, we see in overall no significant improvement or deterioration of survival prediction with merged data sets as compared to individual data sets. This apparently was due to the fact that a few genes with strong prognostic power were not available on all microarray platforms and thus were not retained in the merged data sets. Surprisingly, we found that the overall best performance was achieved with a single-gene predictor consisting of CYB5D1.

**Conclusions:**

Merging did not deteriorate performance on average despite (a) The diversity of microarray platforms used. (b) The heterogeneity of patients cohorts. (c) The heterogeneity of breast cancer disease. (d) Substantial variation of time to death or relapse. (e) The reduced number of genes in the merged data sets. Predictors derived from the merged data sets were more robust, consistent and reproducible across microarray platforms. Moreover, merging data sets from different studies helps to better understand the biases of individual studies and can lead to the identification of strong survival factors like CYB5D1 expression.

## Introduction

Microarray gene expression data have been integrated to increase statistical power. Increasing sample size is a bottleneck in DNA microarray-based gene expression studies as microarray experiments are time consuming, expensive, noisy and limited to the number of biological samples (particularly, cancer patients). To circumvent this problem, microarray gene expression data sets addressing the same or similar biological questions have been analyzed jointly either by so-called meta analysis [1]–[5], which means integration at the level of results derived separately from individual data sets, or by data merging [6]–[16]. But data integration prior to analysis potentially faces problems related to reproducibility as different microarray platforms use different probes for the same genes and return expression values on different numerical scales.

Some studies combined data sets generated with the same chip [6]–[8], [10], or with the same technology platform but different chips [9], [11]–[14], [17], [17]–[19], or with heterogeneous microarray technologies [15], [16], [20], [21]. The rationale behind combining data sets generated only from the same chip or platform was to avoid the cross-platform bias. As it is difficult to measure absolute mRNA concentrations by hybridization-based expression profiling techniques, one would expect gene-specific systematic differences between expression values obtained with different probes. Systematic differences between published data sets may also result from different pre-processing steps applied by the authors. For instance, expression levels are sometimes expressed as absolute values, sometimes as log ratios with respect to a reference sample. To avoid bias resulting from preprocessing, Reyal et al [6] restricted their studies to data sets generated with the same chip (Affymetrix HG-U133A) for which raw data were available, and re-processed all data sets prior to merging. Other studies used homogeneous (same technology) [8], [9],[11]–[13] or heterogeneous [15], [16], [20] data sets, as pre-normalized in the original studies, and applied a so-called data integration method prior to data fusion.

A data integration method serves to project expression values for the same gene onto comparable scales. Perhaps the simplest way to approximately achieve this goal is Z-score normalization [22]. More advanced methods attempt to match data-set specific parameters of the expression value distributions between input sets. Data integration methods that have been used in similar studies before include: Distance Weighted Discrimination (DWD) [23], [11], [14]–[16], [24], Combatting Batch effects (ComBat) [25], [12], disTran [26], Median Rank Score (MRS) [20], Quantile Discretizing (QD) [20] or Z-score transformation [22].

Another necessary processing step in data merging consists of mapping microarray features to a catalogue of standard gene names. This in turn will result in the definition of the subset of common genes to be retained in the merged data set. Here, the term microarray feature refers to a single hybridization probe, or a set of probes, for which the platform returns a single expression value. Commercially available microarrays often contain multiple features for the same gene. What makes the merging of data sets non-trivial is that different platforms refer to the same genes by different names. Note further that for the reasons outlined above, merging of data sets usually leads to a substantial reduction in the number of genes considered for downstream analysis. Important genes included in only a part of the input data sets may be lost.

Some studies [10], [15], [17], [20] used UniGene ID [27] to identify common genes between different data sets whereas other studies employed different databases such as RefSeq [28], [17] or Stanford Source database (http://source.stanford.edu) [14], [16] to match probes/probe sets to genes. Note further that some research teams used directly probe/clone identifiers [14] or probe set IDs [6]–[8], [12], [13] when merging only cDNA or Affymetrix data set collections, respectively. The latter studies might have preferred not collapsing features (Probe/probe set) into genes in order to keep the same annotation as other studies to validate the same features. An additional reason to keep original feature IDs is to preserve a large number of features rather than a a smaller number of genes to make biological/statistical inferences. Sohal and coworkers [17] used both UniGene ID and RefSeq ID to make a comparison of common genes. They concluded that using UniGene IDs achieved slightly better results than using RefSeq IDs, with a small margin. In this study, we used our own resource CleanEx [29] for mapping microarray features to gene names, a database specifically developed for this purpose.

While some research projects merged the gene expression values in their original continuous representation [6], [11], [12], [14], [15], some other studies combined the ranks of gene expression values [7]–[9], [20], [30] which are independent from normalization. In these studies, ranking was used to predict a categorical outcome. Note that ranking methods replace the continuous values by discrete integer values which influences the choice of data integration method. While DWD and ComBat preserve the original representation of data, MRS, QD and disTran transform the data representation into discrete values.

In previous studies, merging data sets was applied to derive a robust gene signature prognostic of survival time (Overall Survival (OS) [11], or prognostic of survival outcome discretized into two [6]–[10], [13], [20], or more categorical values [12], [14]–[16], [20], [24], or diagnostic of tumor subtypes [14]–[16], [24], or predictive of treatment response [12]. The gene signatures were built by a supervised machine learning algorithm like Support Vector Machines (SVM) [7], [10], [13], [20]–[22] or unsupervised classification methods like clustering [14]–[16], [24] or statistical method such as Cox regression model [11] and likelihood ratio test [9]. Such gene signatures consist of a list of genes, usually associated with weights that are used to compute a predictive score.

In previous tests, the potential advantage of data merging was evaluated by means of a quantitative rate of correct classification or validation of previous results. Note however that different studies used different performance measures (sensitivity, specificity, Area Under the Curve (AUC), percentage of concordance in classification, etc) to this end.

Gene signatures are also evaluated in terms of “robustness” and “reproducibility”. Robustness is related to the sample size of the training set from which a gene signature is built and the size of the testing set on which it is validated. A predictor generated from a small training set could have a high prediction accuracy on the training set but may lose generalization power when it is validated on an independent testing set. Moreover, performance estimates obtained with a small testing set have high statistical error, i.e. they come with a large confidence interval. On the other hand, reproducibility [31] means the convergence of results obtained from replicate experiments, possibly carried out in different labs and relying on different technologies. Reproducibility is assessed by cross-data set validation, i.e. the evaluation of a gene signature derived from one data set, with a testing set originating from another study.

In this work, we analyzed the potential benefits of merging data sets for prognostic application in breast cancer diagnosis. Contrary to related work, we did not discretize the clinical follow-up information into good and poor outcome classes, a practice which results in loss of information. Instead, we directly used censored survival data to derive a gene signature that allows for the computation of a risk score from a patients expression profile. The risk score was based on the Cox proportional hazard model, and expected to be inversely related to the time to death or relapse.

**Figure 1:**
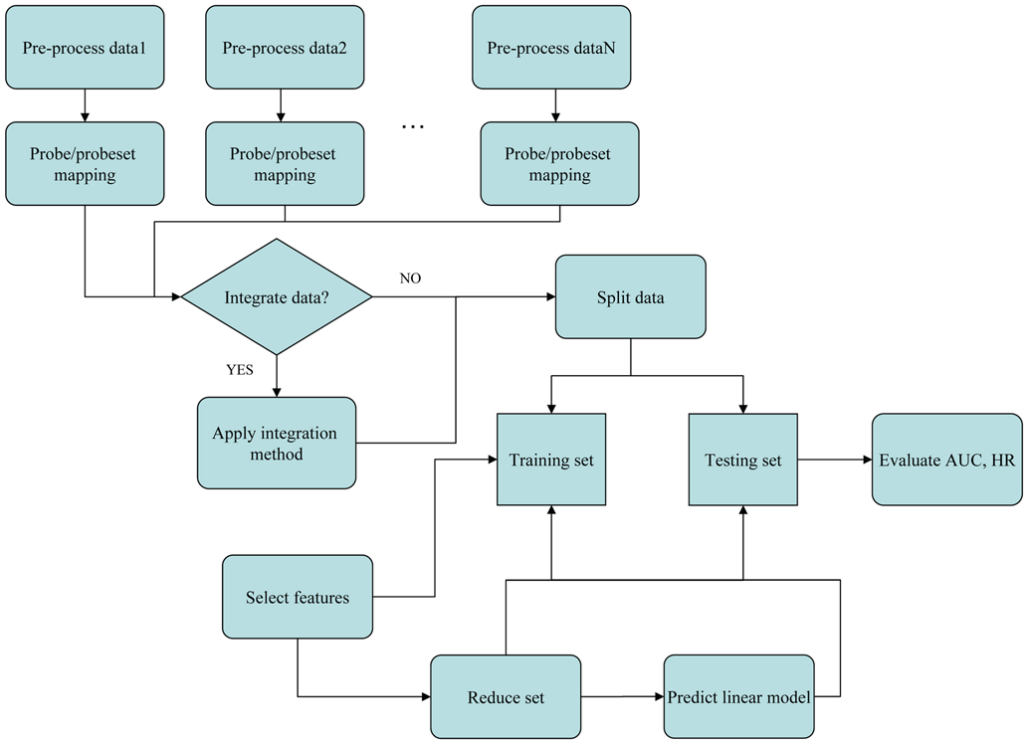
The flowchart of this study.

The basic design of our study is as follows (Figure 1). We used eight breast cancer microarray data sets from eight different studies (Table 1). Each set had clinical follow-up information in form of censored time to event data, the event being either “overall survival” (OS) or “relapse-free survival” (RFS) or both. The goal was to extract a gene signature from a training set that can be used to predict disease outcome for patients in the testing set. The gene signature (predictor) we used consisted of a set of genes plus corresponding Cox coefficients derived by univariate fitting of the expression values to the survival data on the training set. Gene signatures were built from the individual or merged data sets. The accuracy and robustness of prediction were evaluated by 10-fold cross-validation. Reproducibility as defined above was analyzed by training a signature from one or several complete data sets and testing its performance on complete independent validation sets.

**Table 1:** Survival breast cancer datasets with OS and RFS endpoints.

Data set	Platform	Pre-normalization	Gene nb	Sample size	Ref.	Treatment	Survival outcome
GSE3143	Affymetrix, HG-U95A	MAS5.0	8660	158	Bild 06 [55]	Unknown	OS
GSE1456A&B	Affymetrix, HG-U133A&B	MAS5.0, global mean	15848	159	Pawitan 05 [56]	Adj. Chemotherapy (incl. Tamoxifen)	OS, RFS
GSE4335	cDNA	Scaling	12793	122	Sorlie 03 [57]	Neoadj. Chemo/chemo (Tamoxifen)-82 patients	OS, RFS
GSE1992	Agilent	LOWESS	15528	170	Hu 06 [15]	Treated	OS, RFS
Vijver	Agilent	Scaling	13628	295	Van de Vijver 02 [36]	Chemo/hormonal therapy (90 patients)	OS, RFS
GSE2990	Affymetrix, HG-U133A	RMA	12010	189	Sotiriou 06 [46]	Tamoxifen (64 patients)	RFS
GSE2034	Affymetrix, HG-U133A	MAS5.0	12010	286	Wang 05 [38]	None	RFS
GSE4922A&B	Affymetrix, HG-U133A&B	MAS5.0, global mean	15848	289	Ivshina 06 [58]	Systemic/endocrine therapy (147 vs. 66 patients)	RFS
Merged OS	Affymetrix, Agilent, cDNA		7049	849			OS
Merged RFS	Affymetrix, Agilent, cDNA		9181	1324			RFS

Gene nb refers to the number of genes. MAS 5.0 refers to Affymetrix Microarray Suite version 5.0 and LOWESS stands for LOcally WEighted Scatter plot Smoothing and RMA for Robust Microarray Analysis, respectively. Adj. stands for adjuvant and chemo for chemotherapy. Merged OS refers to merged data sets with Overall Survival endpoint and Merged RFS refers to the merged data sets with Relapse Free Survival endpoint. The expression values of dual channel data were already 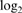-transformed. Among the data sets generated by Affymetrix, the absolute intensity values of GSE3143, GSE2034 and GSE2990 were 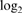-transformed for this study as the rest of Affymetrix data sets were already 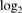
- transformed by the authors.

Data sets were merged in their original (continuous) numerical representation using two different data integration methods: (i) ComBat [25] and (ii) Z-score normalization. Two signature performance measures were computed in each experiment: (i) time dependent Receiver-Operator Characteristic Area Under the Curve (ROC-AUC) and (ii) the hazard ratio (HR) of the predicted risk scores relative to the survival data in the testing set. Note that the latter required stratification of the testing set patients in a high and low risk groups.

In total, we analyzed 1324 breast cancer samples from public data sets generated with three microarray technologies (cDNA, Agilent, Affymetrix). To the best of our knowledge, this study is the largest one evaluating the potential benefits of data merging in a quantitative OS/RFS patients risk prediction framework.

## Results

### Verification of Data Integration

To assess the removal of microarray bias effect across data sets, Principal Component Analysis (PCA) and hierarchical clustering were applied to the data sets after the application of data integration methods.

**Figure 2:**
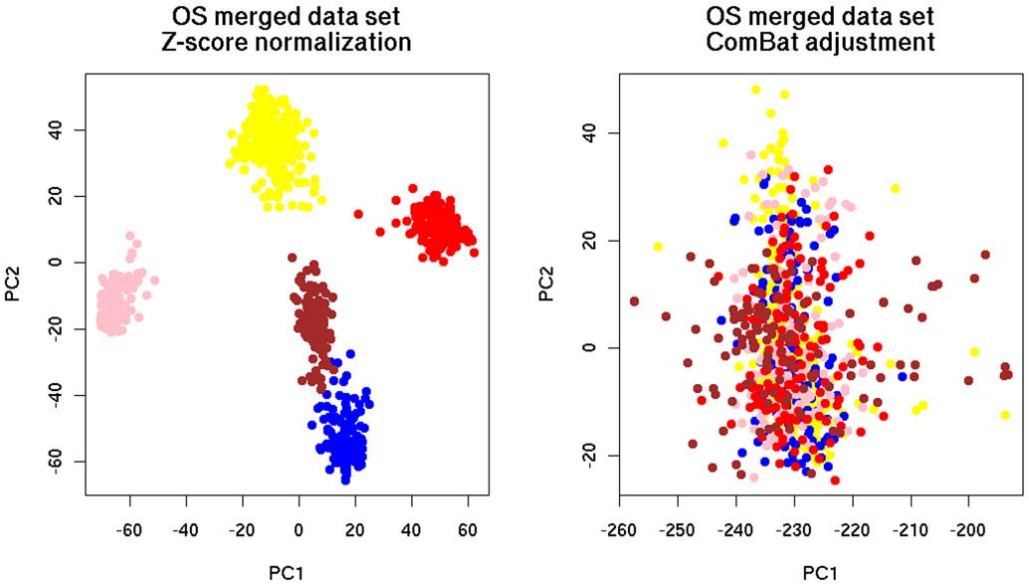
Distribution of the merged breast cancer data sets with OS after the application of PCA. Color legend of data source: yellow: Vijver, blue: GSE1992, pink: GSE1456, red: GSE3143, brown: GSE4335.

**Figure 3:**
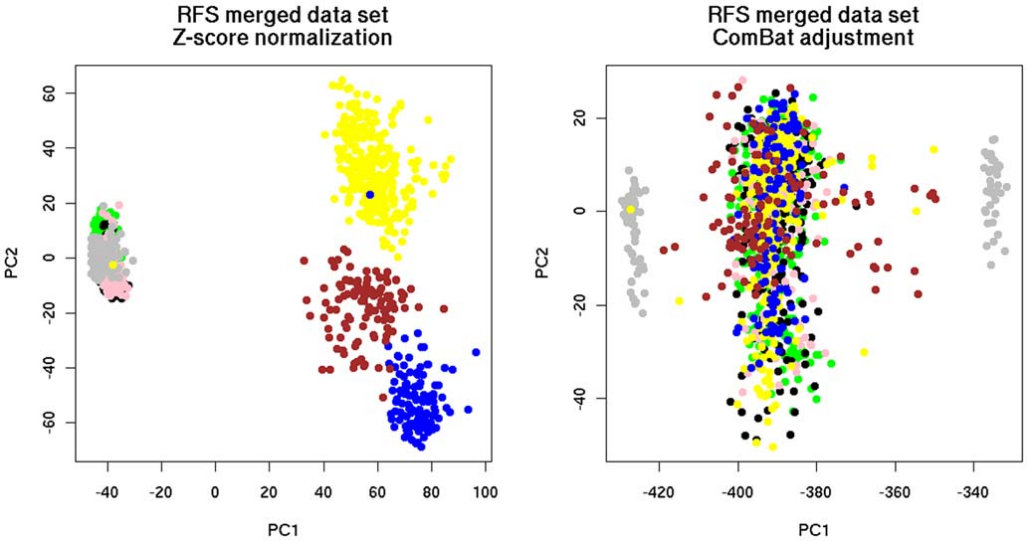
Distribution of the merged breast cancer data sets with RFS after the application of PCA. Color legend of data source: yellow: Vijver, blue: GSE1992, pink: GSE1456, grey: GSE2990, brown: GSE4335, black: GSE4922, green: GSE2034.

**Figure 4:**
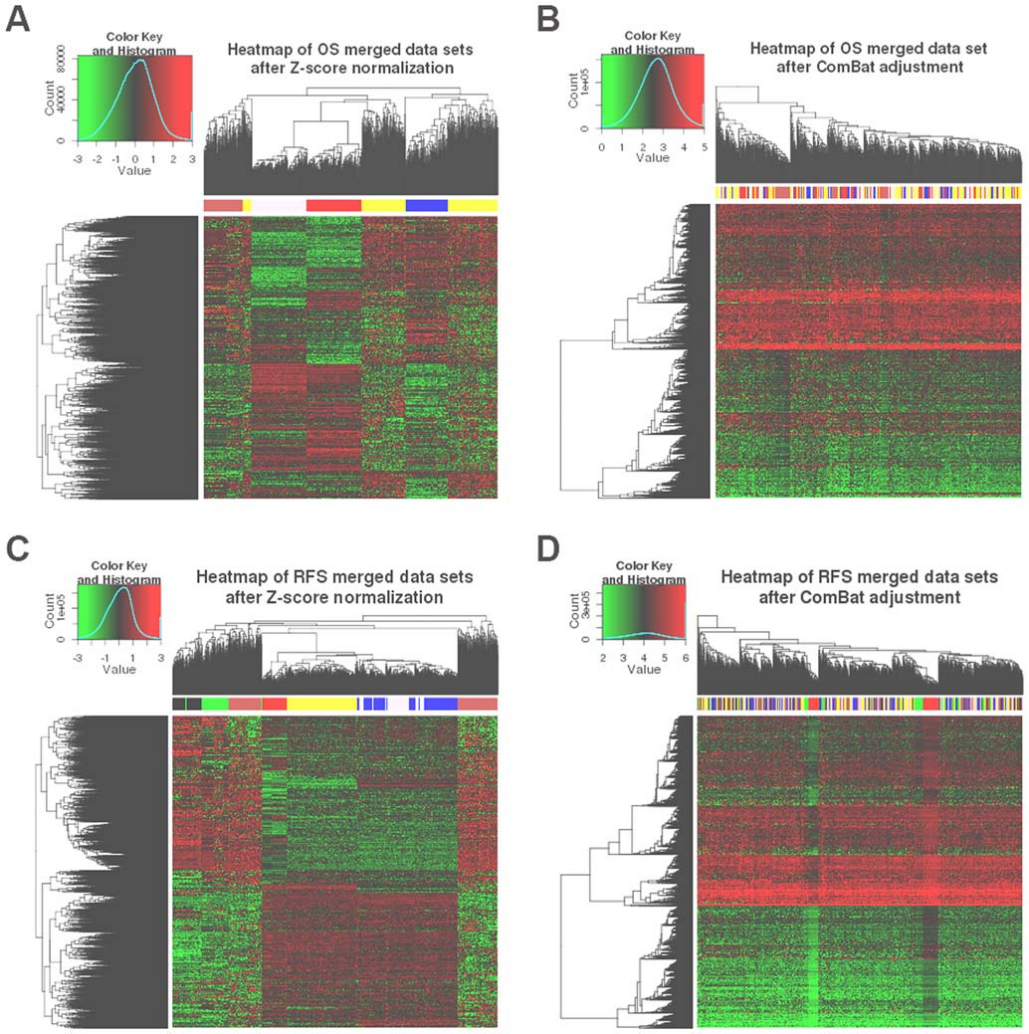
Heatmaps of the merged breast cancer data sets for all genes. (A) Z-score normalization with OS endpoint, (B) ComBat adjustment with OS endpoint, (C) Z-score normalization with RFS endpoint, (D) ComBat adjustment with RFS endpoint. Genes are presented in rows and samples are illustrated in columns. Color legend of data source for Figure 4A and Figure 4B: yellow: Vijver, blue: GSE1992, pink: GSE1456, red: GSE3143, brown: GSE4335. Color legend of data source for Figure 4C and Figure 4D: yellow: GSE2034, blue: GSE4922, pink: GSE1456, red: GSE2990, brown: Vijver, black: GSE1992, green: GSE4335.

The results of these tests are shown in Figure 2, Figure 3 and Figure 4. In all figures, samples from the same source are represented by the same color. Samples from different sources are represented by different colors. In the heatmaps, green color of pixels illustrates low expression level and red color depicts the high expression level of the genes, respectively.

For the verification of data integration by PCA, the merged data sets were projected on the planes defined by the first two principal components (PCs). The purpose was to demonstrate intermixing of samples from different sources. As can be seen, ComBat was successful in removing dataset-specific biases (Figure 2, Figure 3) since the samples of the merged data set integrated with ComBat were better intermixed than the samples merged with Z-score normalization.

The PCA results were confirmed by clustering. Figure 4 presents the results for the datasets with OS and RFS events annotation, respectively. The samples were grouped by data source when they were normalized by Z-score normalization (Figure 4A, Figure 4C). Here, genes are presented in rows and samples are organized in columns. After the application of ComBat, the influence of data source on grouping was significantly reduced (Figure 4B, Figure 4D).

### Assessment of Gene Signatures

#### Evaluation in 10-fold Cross Validation

**Figure 5:**
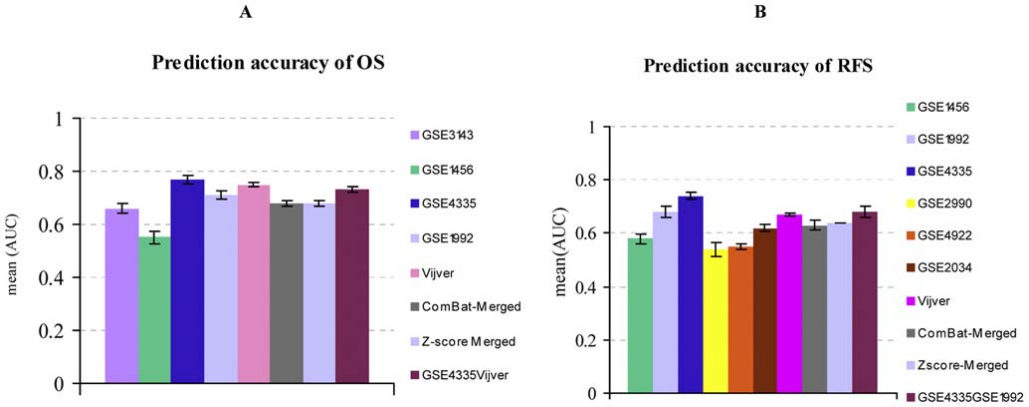
The prediction accuracy of the gene signatures generated from the breast cancer merged and single data sets. The results present the average of the Area Under the Curve (AUC) over 10-fold cross validation. The error bars represent the standard deviation of AUC over 10 iterations. Figure 5A: GSE3143: 0.66+/−0.03, GSE1456: 0.55+/−0.04, GSE4335: 0.77+/−0.02, GSE1992: 0.70+/−0.05, Vijver: 0.75+/−0.01, ComBat-merged: 0.68+/−0.03, Zscore-merged: 0.68+/−0.01, GSE4335Vijver: 0.73+/−0.02. Figure 5B: GSE1456: 0.58+/−0.02, GSE1992: 0.67+/−0.03, GSE4335: 0.74+/−0.03, GSE2990: 0.54+/−0.04, GSE4922: 0.55+/−0.02, GSE2034: 0.62+/−0.02, Vijver: 0.67+/−0.01, ComBat-merged: 0.63+/−0.01, Zscore-merged: 0.64+/−0.00, GSE4335GSE1992: 0.68+/−0.02.

**Figure 6:**
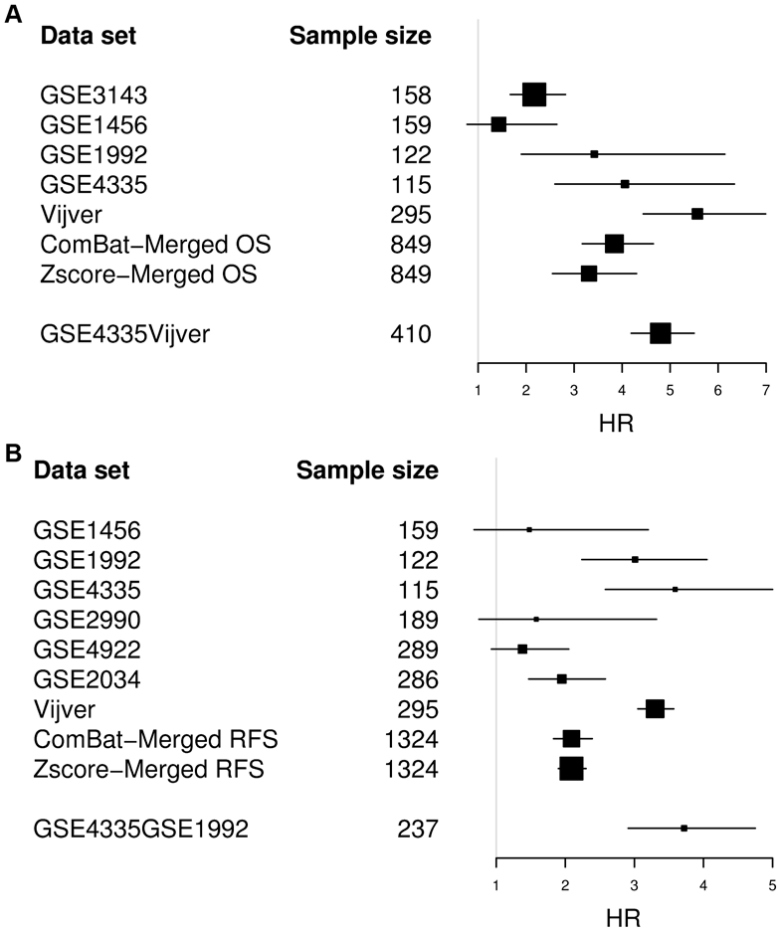
HR of the gene signatures derived from the merged and single breast cancer data sets. Figure 6A: GSE3143: HR = 2.17, CI = 1.67–2.82, GSE1456: HR = 1.43 CI = 0.77–2.64, GSE1992: HR = 3.42 CI = 1.90–6.14, GSE4335: HR = 4.06 CI = 2.60–6.34, Vijver: HR = 5.57 CI = 4.44–6.99, ComBat-merged OS: HR = 3.84, CI = 3.17–4.65, Zscore-merged OS: HR = 3.31, CI = 2.55–4.30, GSE4335Vijver: HR = 4.80, CI = 4.19–5.50. Figure 6B: GSE1456: HR = 1.48, CI = 0.68–3.20, GSE1992: HR = 3.01, CI = 2.24–4.05, GSE4335: HR = 3.59, CI = 2.58–5.00, GSE2990: HR = 1.58, CI = 0.75–3.32, GSE4922: HR = 1.38, CI = 0.93–2.05, GSE2034: HR = 1.95, CI = 1.47–2.58, Vijver: HR = 3.30, CI = 3.05–3.57, ComBat-merged RFS: HR = 2.09, CI = 1.83–2.39, Zscore-merged RFS: HR = 2.09, CI = 1.83–2.39, GSE4335GSE1992: HR = 3.72, CI = 2.91–4.75.

The major goal of our study was to assess the benefits of data merging with regard to disease outcome prediction. To this end, we derived gene signatures based on Cox regression from the individual and merged datasets as described in the Methods section. The results of these tests can be summarized as follows. For the data sets with OS endpoint (Figure 5A, Figure 6A), the average AUC over 10-fold cross validation (CV) remained comparable between the merged and single data sets within the limits of the respective standard deviations (ranging from 0.01 to 0.05). Although neither the prediction power (AUC) nor the hazard ratio (HR) with the merged data sets increased significantly, the HR of the merged data sets had a more robust (shorter) asymptotic 95% confidence interval (CI).

We compared two data integration methods: (i) ComBat and (ii) Z-score normalization as described under Methods. Based on PCA and clustering results, we observed that ComBat was more efficient in inter-mingling the samples. We therefore expected that ComBat would outperform Z-score normalization. However, we noticed that the two methods performed about equally well. The AUC values generated from the merged data sets based on the two methods were identical. The HR however is slightly higher for ComBat (ComBat: HR = 3.84, CI = 3.17–4.65, Z-score: HR = 3.31, CI = 2.55–4.30, Figure 6).

One possible explanation for this apparent contradiction would be that the first two principle components and the clustering trees obtained after data integration reflect biologically irrelevant technical variance not related to data source. We tested this hypothesis by fitting the first two principal components obtained after the application of ComBat and Z-score normalization to the merged survival data. We obtained the hazard ratios of 1.40 and 0.79 for PC1 and PC2, respectively, after Z-score normalization (P-values 6.3e-07 and 1.3e-03), and 0.97 and 1.54 for the ComBat merged data sets (P-values 7.3-e01 and 3.7-e14). While these values do not precisely confirm our hypothesis, they indicate that the first two principal components capture the variance related to survival about equally well in both cases.

We observed similar trends when looking at the distribution of Estrogen Receptor positive (ER+) and Estrogen Receptor negative (ER-) samples in the clustering diagrams. There we saw both after the application of ComBat and Z-score normalization many small clusters of ER- samples (the minority class) spread over the entire range of the tree (data not shown). While these findings conciliate the results from data integration verification with those from gene signature evaluation, they also reveal the limited usefulness of the data intermingling test, which in this case provides a misleading picture of the variance retained after data integration.

Noting that the gene signatures built from subsets of GSE4335 or Vijver showed higher prediction accuracies in cross-validation than the gene signature built from the merged data set, we investigated how the performance could possibly be improved by selective data integration. With regard to both OS and RFS results, we made the following general observation: (i) The prediction accuracy obtained with a signature from a merged data set lies between the accuracies of the signatures derived from the component data sets. (ii) There are marked differences in performance of signatures from individual data sets, possibly reflecting the quality of the data or the diversity of the analyzed patient cohorts. Overall, these observations are in agreement with results from similar tests where performance was measured by a binary classification assay [7], [20], [30].

Taking into account this observation, the data sets with the highest prediction accuracies were merged in different combinations in the hope to improve the overall prognostic power of a signature based on a merged data set. Firstly, the data sets GSE4335 and Vijver were merged and adjusted by a data integration method (ComBat or Z-score normalization), resulting in a predictor with an average AUC of 0.73+/−0.01, HR = 4.8 (CI = 4.19–5.50). Adding the data set GSE1992 reduced the prognostic power by 0.01 only (average AUC = 0.72, HR = 3.84, CI = 3.17–4.65). Combining the data sets GSE4335 and GSE1992 resulted in a mean AUC of 0.70 (+/−0.02) and an HR of 3.35 (CI = 2.55–4.30). The AUC obtained from the combination of GSE4335 and GSE1992 is comparable to the result obtained by combining GSE4335 and Vijver but the HR decreased by 1 unit. Adding the Vijver data set to GSE4335 and GSE1992 resulted in a similar prediction accuracy of mean AUC 0.72+/−0.01 but a higher HR = 4.53, CI = 4.03–5.09. These results show that the performance of the merged data set composed of two data sets was as good as the merged data set composed of five data sets. Interestingly, these pairs of data sets (GSE4335 and Vijver or GSE4335 and GSE1992) were generated by different gene expression profiling technologies, indicating that platform-specific biases were not a major obstacle to data merging. Importantly, these results suggest that the potential added-value gained by including a specific data set in a merged data set is predictable from its cross-validated performance measured in isolation.

Taken all these together, however, the improvement seen with selectively merged data sets is relatively modest as compared to the prognostic power generated from the merged data sets composed of all OS single data sets. Nevertheless, this improvement is remarkable if one takes into account that the selectively merged data set only contains about one-third (237) or half (410) as many samples as the complete merged data set (849).

In the case of RFS (Figure 5B), the merged data sets adjusted by ComBat and Z-score normalization outperformed the single data set GSE2990 by 0.07 AUC. Compared to the other single data sets, the results generated from the merged data sets remained equal or lower by 0.00–0.06 AUC units. Here, the combination of the data sets GSE4335 and GSE1992 had a better prediction accuracy with a mean AUC of 0.68+/−0.02 and an HR of 3.72 (CI = 2.91–4.75) (Figure 6B), outperforming by 0.05 the prediction accuracy generated from the 1324-sample merged data sets. Adding the Vijver data set resulted in a similar prediction accuracy with a mean AUC of 0.66+/−0.01 and HR = 3.05 CI = 2.24–4.05.

Comparing the performance results of the merged data sets, we observe that none of the merged data sets was more successful in prediction performance than the best individual data sets. Again ComBat and Z-score integration produced similar results, with a slight advantage of the latter method in this case.

To determine the significance and stability of the selected gene signatures, we were then interested to investigate if the top 100-ranked genes would still be selected if a P-value cut-off of 0.05 after Bonferroni correction [32] was applied. We noticed that all the top 100-ranked genes derived from the merged and the Vijver data set had a significant adjusted Cox P-value. This was not the case for the 100-gene signatures built from any of the other single data sets. The 1-gene signature derived from GSE4335 (see below) also systematically had a significant adjusted Cox P-value. The adjusted P-values of the genes contained in the signature appeared to relate to the robustness of the gene signatures rather than to the prediction accuracy measured in cross-validation (compare for instance to the prediction accuracies of GSE4355 to ComBat- or Z-score-Merged in Figure 5A). Our results are in agreement with Van Vliet and colleagues' findings [7].

Regarding the two performance measures used, it is noteworthy that HR was not always directly related to the prediction accuracy expressed as AUC [33]. For example, the gene signature derived from GSE1992 showed a prediction accuracy of 0.68 with an HR of 3.06 in one iteration of CV, and a prediction accuracy of 0.63 with an HR of 3.05 in another iteration.

#### Validation on independent data sets

To assess the reproducibility of the gene signatures' performance derived from the merged data sets, the prediction accuracy was evaluated in a leave-one-data set-out manner (section Bias Estimation). In each step, one complete source data set was set aside as testing set while the predictor was built from the merged remaining sets. In parallel, we carried out pair-wise tests, using one source data set as training and another one as testing set. Table 2, Table 3 and Table S1 to S6 summarize the results of these evaluations with respect to the two clinical endpoints, OS and RFS.

**Table 2:** Cross-data set performance of the breast cancer predictors trained on the individual and combined data sets (by ComBat) with respect to OS.

	GSE1456	GSE1992	GSE4335	Vijver	GSE3143	Merged-ComBat 
GSE1456	NA	**0.67**	**0.70**	**0.77**	**0.71**	**0.75**
GSE1992	**0.62**	NA	0.57	0.56	**0.61**	**0.62**
GSE4335	**0.62**	**0.68**	NA	**0.73**	**0.66**	**0.70**
Vijver	**0.77**	**0.73**	**0.70**	NA	**0.68**	**0.77**
GSE3143	**0.67**	0.47	0.54	0.56	NA	**0.62**

Significant AUC (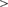
0.6) are shown in bold. The training sets are listed in the column header and the testing sets are indicated in the row header of the table.

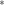
indicates that the predictor was trained from all data sets except the testing set. NA stands for Not Available.

**Table 3:** Cross-data set performance of the breast cancer predictors trained on the individual and combined data sets (normalized by Z-score normalization) with respect to OS.

	GSE1456	GSE1992	GSE4335	Vijver	GSE3143	Merged-zscore 
GSE1456	NA	**0.64**	**0.68**	**0.76**	**0.68**	**0.77**
GSE1992	0.56	NA	0.59	0.56	**0.63**	**0.60**
GSE4335	**0.65**	**0.66**	NA	**0.69**	**0.76**	**0.70**
Vijver	**0.75**	**0.72**	**0.69**	NA	**0.71**	**0.75**
GSE3143	**0.62**	0.51	0.50	0.56	NA	**0.60**

Significant AUC (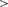
0.6) are shown in bold. The training sets are listed in the column header and the testing sets are indicated in the row header of the table. Merged-zscore refers to the merged data set composed of the individual data set normalized separately by Z-score normalization.

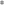
indicates that the predictor was trained from all data sets except the testing set. NA stands for Not Available.

In this test, merging data sets improved overall survival prediction and risk association. This interpretation is based on the fact that for a given testing set, the predictor built from the merged training set outperformed on average 3 out of 4 predictors built from the individual sets. With respect to OS, the survival prediction based on Z-score normalization was higher than the prognosis accuracy obtained with ComBat. A partial improvement of the association of the 100-gene signatures with OS (measured by HR) by merging data sets can also be observed in Table S1 and Table S2. In this analysis, ComBat outperformed Z-score normalization.

The partial improvement of survival prediction and risk association that were observed for OS endpoint were also obtained with respect to RFS. Between the merged data sets, Z-score normalization provided higher results than ComBat even though the difference is not significant (see supplementary information file, Table S3 to S6).

### Overlap between gene signatures

Previous reports pointed out limited overlap between gene signatures (see discussion in [34], [35]). We were wondering whether this observation could be confirmed by our experiments, and therefore went on to compare the different gene signatures obtained in the previous tests to each other. Unsurprisingly, the top 100-ranked genes generated from different single data sets had no or poor overlap with each other and with the merged data sets (data not shown). Reportedly strong prognostic markers like ESR1 and GATA3 were not systematically selected from all data sets. For example, ESR1 and GATA3 were in the gene signature generated from GSE4335 but not in the gene signatures derived from GSE1456 or GSE1992.

The genes selected from the merged data sets based on RFS were matched to the 70-gene signature published by Vijver et al. 2002 [36]. Note that the 70-gene signature mapped to only 57 genes (CleanEx release of the 3rd September 2007). Only eight of these genes (C16orf61, CENPA, DTL, MELK, NDC80, NUSAP1, ORC6L, PRC1) were also found in the gene-signature derived from the merged data sets adjusted by ComBat. Even fewer (four) common genes (CENPA, HRASLS, PECI, PRC1) were found in the merged data set normalized by Z-score normalization. This small overlap is in agreement with reported observations on breast cancer gene signatures. It is furthermore expected from theory and simulations [35], [37].

### Factors limiting the success of data merging

To find out why data merging did not lead to an improvement of performance, a test series were carried out to optimize the gene signature size for the selected individual and merged data sets adjusted by ComBat or Z-score by varying the number of genes from 1, 5, 10, 20, 50, 150, 200, 500 to 1000. We selected for this test those individual sets, which had annotations for both clinical endpoints, OS and RFS. Overall, this analysis confirmed that in most cases the size of 100 was a near-optimal choice for survival prediction by a Cox regression model (supplementary information Figure S1). Based on over-fitting considerations, it was expected that the merged data sets containing more samples would tolerate larger gene signatures. However, this conjecture was not (or not clearly) supported by the results.

### Case of the gene CYB5D1

#### New findings on CYB5D1

On comparing the results obtained for AUC and HR from the individual data sets with the signatures of variable size, it was surprising to observe the large variation in the performance profiles. Most strikingly, with the data set GSE4335, the best performance was obtained with a signature consisting of a single gene. With an AUC value of 0.8 for OS prediction, this was the overall best performance registered in this study. It has to be remembered in this context that the performance was measured in cross-validation. It was thus not clear whether in the case of a single-gene signature the same gene or different genes were selected in each fold of cross-validation. In principle, the prediction accuracy could reflect the average performance of different genes selected in successive iterations. Looking at the results after each fold of cross-validation, we noticed that the gene CYB5D1 (cytochrome b5 domain containing 1) was selected in 95% of the cases. The Cox coefficients obtained for this gene were consistently negative, indicating a beneficial effect on survival. With respect to RFS endpoint, this gene was selected in 78% of the cases and the prediction power of the one-gene signature was found to be 0.73.

We were not the first to find that a 1-gene signature had an equally good or stronger prognostic power than a gene signatures with a higher number of genes. Haibe-Kains and colleagues [3] previously found the proliferation gene AURKA to be a strong survival predictor at least as good as any composite gene predictor based on advanced machine-learning techniques.

The excellent performance of one gene in survival prediction immediately raises the question whether this gene was included in all studies. If this were not the case, then the gene would not be present in the merged data set, which could explain the rather disappointing prediction accuracy figures obtained after merging. Indeed, this gene was absent in a number of data sets. In one other data set where it was present, its performance as a single-gene signature in risk prediction was consistently high (GSE1992, AUC = 0.68, HR = 3.24, P-value = 0.008, CI = 1.36–7.72). The influence of this gene could also explain the good performance obtained after merging selectively the sets GSE4335 and GSE1992 (see above). A possible reason that adding the Vijver data set did not improve the prediction accuracy might be due to the fact that this gene was absent in the original Vijver study. As expected, the Cox coefficient of this gene was negative for all data sets in which it was present, meaning low risk associated with high expression levels. The case of CYB5D1 suggests that the absence of key risk or survival genes from some microarray platforms may in general be a major limiting factor in data merging, demonstrated here on the specific example of survival prediction for breast cancer patients.

### Known findings on CYB5D1

The full name of CYB5D1 is “cytochrome b5 domain containing 1”. It is noteworthy that the gene is absent from the well known prognostic gene signatures published by van de Vijver et al. [36] and Wang et al. [38] even though this is not surprising as the gene was not present on the gene expression profiling platform used in these studies. We wanted to know whether this gene was previously found as a survival gene in similar studies. An initial search with gene symbol CYB5D1 was unsuccessful. However, using UniGene ID Hs.27475 [27], we found a few earlier cancer studies where this gene was selected as a differentially regulated gene.

Jenssen and colleagues [39] found Hs.27475 associated to breast cancer survival. In their study, they used the Sorlie data set [40], a subset of GSE4335 analyzed in this work. Using a Log-rank test applied to discretized survival data, they identified 95 genes positively or negatively correlated with survival (P-value
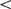
0.05, with Bonferroni correction [32]). In their gene list ranked by P-value, Hs.27475 appeared at rank 11 (rank 6 if positively correlated genes are considered only) with P-value 9.4e-10.

The expression of Hs.27475 was also found to be positively correlated (Pearson correlation of 0.58) with the expression of Estrogen Receptor 1 (ESR1) [41]. In this study performed by Mackay et al., paired biopsies from 34 ER positive breast cancer patients, taken before and after treatment were analyzed with cDNA microarrays. The patients were treated with aromatase inhibitors like anastrozole and letrozole that are suppressants of estrogen synthesis. It needs to be pointed out that the main goal of this study was to identify estrogen target genes, not genes that correlate with ESR1 expression. In this respect, Hs.27475 was not among the 1,395 most upregulated or the 1,264 most down-regulation genes found by the analysis of the paired samples.

Hs.27475 was noticed to be about two-fold downregulated in two other types of cancer, primary colon and primary rectal carcinoma [42]. In this study, the colon data set was composed of 73 tumors of locally advanced colon carcinomas that were profiled on oligonucleotide microarrays containing 21543 features. The rectal data set was taken from a previous study [43] in which 29 rectal carcinoma and 20 normal mucosa samples were analyzed on a oligonucleotide microarray containing 22,231 features. Hs.27475 was down-regulated at P-value
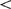
0.0001 in the two data sets. Note further that this gene was among 490 common genes found to be deregulated (up or down) in both tumor types. The down-regulation of CYB5D1 colon cancer is consistent with its role of a survival gene in breast cancer.

It may also be worthwhile to mention that CYB5D1 belongs to the same protein family as Progesterone receptor membrane component 1 (PGRMC1), a well known player in the breast cancer field [44]. However, this gene shows opposite behaviour in most respects. It is over-expressed in cancer, particularly in ER negative breast cancer, and correlates with poor prognosis as it makes cells resistant to chemotherapeutic drugs [44]. However, the findings related to PGRMC1 may help to elucidate the molecular mechanisms through which CYB5D1 reduced the malignant potency of breast cancer cells. PGRMC1 binds to and presumably regulates the enzymes of the P450 family, whose activity may interfere with drug resistance or intracellular signalling pathways [44].

In summary, the findings summarized above indicate that CYB5D1 is an important but currently not well known survival gene in breast cancer with potential diagnostic and therapeutic value.

### Survival analysis of patients information and tumor characteristics

Previous breast cancer studies attempted to improve survival prediction by integrating clinical variables with microarray gene expression data [15], [36], [38], [45]–[48]. In these studies, the clinical variables and the gene signatures were fitted simultaneously in a multivariate Cox regression model to determine their adjusted HR. The purpose was to find out if the expression data derived gene signatures had true added value with regard to clinical risk factors or whether the association of the gene signature was mediated entirely by clinical variables.

Five patients characteristics and clinicopathological parameters such as age, tumor grade, tumor size, estrogen receptor (ER) status and lymph node status that were available in five data sets were used here (Table 4). We also carried out tests in these settings with the specific goal to find out whether the gene signatures derived from the merged data sets had more clinical variable-independent predictive power than the gene signatures derived from the individual sets. Details of the protocol are described under Methods section.

**Table 4:** Clinical data of the single and merged breast cancer data sets.

Data set	Grade1	Grade2	Grade3	Size1	Size2	Size3	Size4	Age( 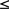 50y)	Age( 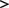 50y)	ER+	ER-	LN+	LN-
GSE4335	11	49	53	6	13	62	32	34	81	82	31	79	34
GSE1992	12	43	63	30	59	21	11	55	67	70	50	71	50
Vijver	75	101	119	155	140	0	0	264	31	226	69	144	151
GSE2990	64	48	55	103	80	4	0	62	125	147	34	30	153
GSE4922	68	126	55	165	84	0	0	54	195	211	34	81	159
Merged OS	98	193	235	191	212	83	43	353	179	378	150	294	235
Merged RFS	193	339	325	340	382	114	43	635	247	661	212	550	327

The number of patients by clinical variables is presented in each cell of the table. ER+ = Estrogen Receptor positive, ER- = Estrogen Receptor negative, LN+ = Lymph Node positive, LN- = Lymph Node negative.

As Table 5 and Table 6 show, merging data sets improves the association of the gene signatures with OS compared to only one data set (out of three) and increases the HR of the gene signatures with respect to RFS for four out of five data sets. These results show that integration of data sets improved partially the adjusted HR of the gene signatures. On the other hand, the adjusted HR did not improve after merging for tumor grade or tumor size.

**Table 5:** Adjusted HR of the breast cancer gene signatures and clinical variables with OS endpoint.

Data set	100-gene signature	Grade	Size	ER	LN	Age
GSE4335	2.19 (1.20–3.99)	2.01 (1.80–2.25)	1.31 (1.21–1.42)	0.53 (0.45–0.62)	0.94 (0.92–0.97)	0.68 (0.59–0.76)
GSE4335 1-gene signature	5.28 (2.44–11.41)	2.47 (2.28–2.67)	1.08 (0.96–1.21)	0.41 (0.37–0.45)	0.90 (0.87–0.94)	0.67(0.61–0.76)
GSE1992	1.23 (0.8–1.89)	3.26 (2.96–3.59)	1.95 (1.84–2.07)	0.29 (0.27–0.31)	3.92 (3.36–4.57)	1.20 (0.91–1.58)
Vijver	3.15 (2.15–4.61)	1.48 (1.34–1.63)	1.53 (1.50–1.56)	0.67 (0.63–0.71)	0.97 (0.93–1.01)	1.17 (1.02–1.22)
ComBat merged OS	1.39(0.8–2.42)	2.24 (1.92–2.61)	1.94 (1.90–1.98)	0.51 (0.43–0.61)	1.24 (1.19–1.29)	1.07 (1.00–1.14)
Z-score merged OS	1.36 (1.10–1.68)	1.31 (1.21–1.42)	1.52 (1.49–1.55)	0.84 (0.78–0.91)	1.47 (1.39–1.56)	1.03 (1.00–1.06)

The CI (in parentheses) presents the CI of the geometric mean calculated in 10 iterations of 10 fold cross validation. LN refers to lymph node status and ER to estrogen receptor, respectively.

**Table 6:** Adjusted HR of the breast cancer gene signatures and clinical variables with RFS endpoint.

Data set	100-gene signature	Grade	Size	ER	LN	Age
GSE2990	0.87(0.47–1.61)	1.12(1.08–1.17)	2.79(2.68–2.90)	0.86(0.77–0.97)	0.79(0.73–0.85)	0.59(0.54–0.65)
GSE4922	0.95(0.51–1.76)	1.45(1.34–1.57)	2.12(1.96–2.29)	1.39(1.19–1.62)	1.44(1.38–1.50)	0.91(0.88–0.94)
GSE4335	2.19(1.24–3.87)	1.79(1.51–2.13)	1.61(1.52–1.71)	0.70(0.57–0.86)	0.98(0.92–1.04)	0.63(0.57–0.68)
GSE43351-gene signature	2.49(1.54–4.02)	1.51(1.37–1.66)	2.11(1.88–2.37)	0.57(0.45–0.72)	1.00(0.92–1.08)	0.62(0.56–0.71)
GSE1992	1.78(1.04–3.05)	2.38(2.12–2.67)	1.69(1.62–1.76)	0.53(0.42–0.68)	8.10(7.21–9.10)	0.85(0.76–0.95)
Vijver	2.62(2.17–3.17)	1.20(1.13–1.27)	1.38(1.33–1.44)	1.07(0.99–1.16)	0.91(0.87–0.95)	1.02(0.95–1.13)
ComBat merged RFS	1.33(1.04–1.70)	1.33(1.25–1.41)	1.43(1.4–1.46)	0.79(0.77–0.81)	1.29(1.24–1.34)	0.73(0.69–0.77)
Z-score merged RFS	2.56(1.51–4.33)	1.27(1.09–1.48)	1.57(1.42–1.73)	0.85(0.73–0.99)	1.45(1.34–1.57)	0.99 (0.97–1.01)

The CI (in parentheses) presents the CI of the geometric mean calculated in 10 iterations of 10 fold cross validation. LN refers to lymph node status and ER to estrogen receptor, respectively.

## Methods

Statistical analysis was performed using R [49], version 2.5.1 and BioConductor [50], release 2.0.

### Data sets

The data sets used in this study were pre-normalized in various ways by the authors of the original studies (Table 1). The gene expression data were imported via the CleanEx database [29] which simultaneously provides a mapping of microarray features to gene names. Note that throughout this document, clinical endpoints, clinical outcomes, or prediction outcomes refer to OS and/or RFS.

Time to OS is defined as the time between surgery and death from breast cancer or the last date of follow-up. Time to RFS is defined as the time between surgery and the first recurrence of local, regional or distant-metastatic breast tumor or the last date of follow-up. If OS or RFS time refers to death or recurrence of disease, the corresponding samples have a censoring status of 1 (event happened) or 0 otherwise.

We limited the analysis to 10 years of follow-up as the majority of breast cancer patients had a follow-up of maximum 10 years. All patients having an overall survival or relapse free survival greater than 10 years were censored and their respective clinical endpoint was set to 10 years. All patients in GSE4335 deceased from any other cause than breast cancer were also censored. Fibroadenoma or normal breast samples were discarded from the study (GSE4335, GSE1992). Replicate samples in GSE1992 were eliminated from the study, too. Note that throughout this document, GSE1456 refers to the merged data set of GSE1456A and GSE1456B, GSE4922 to the merged data set of GSE4922A and GSE4922B, respectively. GSE4335 and Vijver are data sets from clinical trials.

The data sets were pre-normalized in the following ways. Global mean normalization was used for GSE1456A&B and GSE4922A&B. The probe set values were natural log-transformed followed by an adjustment of the mean intensity to a target signal value of log 500. The pre-normalization of Vijver data set was performed on an array-by-array basis. Raw intensities from each channel (red or green) were divided by the mean intensity (in linear scale) of the corresponding channel. The other data sets were pre-normalized as described in the legend to Table 1.

Grade, size, ER and lymph node status are discrete variables (Table 4). Grade was originally coded as three types (according to the Tumor-Node-Metastasis (TNM) classification [51]): type 1 (well-differentiated), type 2 (intermediate) and type 3 (poorly-differentiated). ER had binary value, 0 for ER- and 1 for ER+. Lymph node status had also binary value, 0 for negative node and 1 for any number of positive nodes (1 or more). Tumor size was classified into T1, T2, T3 or T4 in the original studies (according to the TNM classification) except for GSE2990 and GSE4922. In these two data sets, tumor diameter was available and converted into discrete classes according to TNM classification. Originally, age had continuous values (in years). Its value was transformed into a binary category: 0 if age is less or equal to 50 years and 1 otherwise.

Samples containing missing values were discarded in the analyses using clinical variables. Note that the five variables, grade, size, ER, lymph node status and age were only available in three data sets (GSE4335, GSE1992 and Vijver) with overall survival endpoint (total of 532 samples) and in five data sets (GSE4335, GSE1992, Vijver, GSE2990 and GSE4922) with relapse free survival outcome (total of 882 samples).

### Pre-processing data

K Nearest Neighbor (KNN) imputation [52] was used to impute missing expression values in the source data sets, using the function impute.knn of the R package impute with default parameters (including k = 10). The probes/probe sets were mapped to Genew gene symbols [53] via CleanEx [29] (release of the 3rd September 2007). When multiple probes/probe sets were mapped to the same gene, the expressions of multiple probes/probe sets were averaged (after KNN imputation).

### Data Integration methods

Z-score normalization (scale function in stats R package) and ComBat [25] were used to adjust the systematic bias of data sets generated by different platforms. Z-score normalization was applied first to the samples and then, to the genes. Z-score normalization was applied after merging if not specified otherwise.

### Feature Selection

Genes were selected based on univariate Cox P-value ranking using the coxph function in survival R package. In this feature selection method, the genes were ranked based on their likelihood ratio P-value and the 100 genes with the smallest P-values were retained as the gene signature if not specified otherwise.

The genes were selected either in Cross Validation (CV) mode or in a training set independent from the testing set. In case of CV, the expression values of the genes were fitted individually to survival data, and ranked by P-values in each fold. Then, the top 100-ranked genes were selected for the gene signature. In case of independent training/testing sets, the genes were selected from the training set.

### Principal Component Analysis

Principal Component Analysis (PCA) was applied using the prcomp function in R stats package with default parameters except that the center parameter was set to FALSE.

### Clustering

The gene expression data were clustered using complete-linkage agglomerative hierarchical clustering based on the Euclidean distance (heatmap.2 function in R gplots package).

### Prediction Method

Patients risk score was calculated as the linear combination of the Cox coefficients estimated from the training set and the corresponding gene expression values (Equation 1).
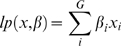
(1)


### Performance Estimation

#### Bias Estimation

The survival predictors were assessed by the following methods:

1. 10-fold cross validation (10fCV) nested in 10 iterations.
2. Leave one data set out: all data sets except one were merged together to form the training set and the left-out set was used as the testing set. This process was iterated until all data sets were used in the training and testing sets.

In 10fCV, the data integration (ComBat or Z-score) was applied separately to the training and testing sets in each cross-validation fold. Note that the training and testing sets were generated by merging and subsequent random splitting. Thus both contained the samples from different sources. Since ComBat needs the source- (batch)-identifiers as input, those identifiers had to be carried through the cross-validation protocol. In case of leave-one-data set-out, Z-score normalization was applied to each data set separately prior to merging.

#### Prediction Estimation

Time-dependent ROC curves [54] were used to evaluate the prediction accuracy at maximum time point for each data set using the nearest neighbor estimator (survivalROC R package). In 10fCV, the accuracy of the prediction of patients survival time (OS or RFS) was represented by the mean and standard deviation (SD).

The association of the gene signatures to survival (OS or RFS) was also measured by a hazard ratio (HR). To this end, the patients of the testing set had to be stratified into predicted high- and low-risk groups. In both 10fCV and independent validation, a testing sample score was considered as high risk, if it was higher than the median score of the training sample and low risk otherwise.

In case of 10fCV, HR was averaged by geometric mean (Equation 2). The 95% CI of the HR over 10 iterations of 10-fold cross validation corresponds to the CI of the geometric mean.
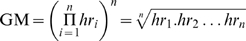
(2)where GM refers to Geometric Mean and 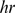 is a vector of 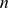 elements.
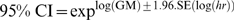
(3)where SE stands for Standard Error.

The gene-signature-based risk scores were also evaluated in the context of clinical parameters, using the same type of cross-validation protocol. The binary risk scores computed for each testing set sample (low/high-risk group as described above) together with the five clinical parameters listed in Table 4 were adjusted to survival data by multivariate Cox regression analysis (using coxph function in survival R package). This fitting procedure returned a hazard ratio for each input parameter. Again, the results of 10fCV were summarized by the geometric mean.

## Discussion

The survival prediction accuracy and prognosis of clinical risk were neither increased nor decreased significantly by merging data sets. This is explained at least in part by the fact that important risk-associated genes were not present in all data sets. Consequently, the heterogeneity of the data sets generated from different laboratories and with different microarray technologies, was not the only, perhaps even not the major limiting factor for improving prediction accuracy by increasing sample size. Substantial variation of time to death or relapse among breast cancer patients and the heterogeneity of breast cancer disease are other constraining factors that nevertheless need to be considered. Moreover, the heterogeneity of patients cohorts in terms of age, lymph node status, tumor grade, tumor size and ER status might negatively affect the accuracy of survival prediction after merging. It is known, for example, that the ER+ patients have good prognosis (long survival) and ER- negative patients have poor prognosis (short survival) in the first five years after the diagnosis or surgery.

Despite the caveats mentioned above, the results show that selectively merging those data sets which give rise to accurate predictors if used alone, can improve the performance. Moreover, our results confirm that the predictors based on large merged data sets are more robust, i.e. their worst performance observed in multiple iterations of cross-validation tends to be substantially better compared to the worst performance of the gene signatures based on the single data sets. This may be viewed as an advantage by itself. In general, the prediction accuracy of the gene signatures derived from the merged data sets remained consistent and reproducible across independent studies. Prediction accuracies measured in cross-validation were extensible to independent testing sets.

The systematic evaluation of predictors built from the single and merged gene expression data sets also led us to the surprising observation that a single-gene signature consisting of CYB5D1 had the highest prediction accuracy and strongest patients risk association in breast cancer, surpassing all gene signatures with different gene size evaluated in this study. CYB5D1 was already mentioned in breast and other types of cancer studies (see results section). The protein encoded by this gene belongs to the same family as Hpr6 (also called PGRMC1) which was found to increase the resistance of tumor cells to DNA-damaging agents. However, the CYB5D1 negatively correlates with a patients risk (found in this study), suggesting that it has the opposite effect. It seems nevertheless plausible that the expression of this gene also interferes with drug metabolism. This hypothesis is compatible with the fact that the strongest predictive power of this gene was seen in a data set primarily composed of patients which received adjuvant chemotherapy before surgery.

Our findings about CYB5D1 call into question the current paradigm that composite gene signatures perform better than one-gene signatures in cancer disease outcome prognosis.

## Supporting Information

Table S1 Cross-data set performance of breast cancer predictors trained on the individual and combined data sets (adjusted by ComBat) with respect to OS. Significant HR (p<0.05) are shown in bold. The training sets are listed in the column header and the testing sets are indicated in the row header of the table. * indicates that the predictor was trained from all data sets except the testing set. NA stands for Not Available.
(0.04 MB PDF)

Table S2 Cross-data set performance of breast cancer predictors trained on the individual and combined data sets (normalized by Z-score normalization) with respect to OS. Significant HR (p<0.05) are shown in bold. The training sets are listed in the column header and the testing sets are indicated in the row header of the table. Merged-zscore refers to the merged data set combined from the individual data sets, each normalized separately by Z-score normalization. * indicates that the predictor was trained from all data sets except the testing set. NA stands for Not Available.
(0.04 MB PDF)

Table S3 Cross-data set performance of breast cancer predictors trained on the individual and combined data sets (adjusted by ComBat) with respect to RFS. Significant AUC (>0.60) are shown in bold. The training sets are listed in the column header and the testing sets are indicated in the row header of the table. * indicates that the predictor was trained from all data sets except the testing set. NA stands for Not Available.
(0.04 MB PDF)

Table S4 Cross-data set performance of breast cancer predictors trained on the individual and combined data sets (normalized by Z-score normalization) with respect to RFS. Significant AUC (>0.60) are shown in bold. The training sets are listed in the column header and the testing sets are indicated in the row header of the table. Merged-zscore refers to the data set merged from the individual data set, each normalized separately by Z-score normalization. * indicates that the predictor was trained from all data sets except the testing set. NA stands for Not Available.
(0.04 MB PDF)

Table S5 HR of breast cancer predictors trained on the individual and combined data sets (adjusted by ComBat) with respect to RFS. Significant HR (p<0.05) are shown in bold. The training sets are listed in the column header and the testing sets are indicated in the row header of the table. * indicates that the predictor was trained from all data sets except the testing set. NA stands for Not Available.
(0.04 MB PDF)

Table S6 HR of breast cancer predictors trained on the individual and combined data sets (normalized by Z-score normalization) with respect to RFS. Significant HR (p<0.05) are shown in bold. The training sets are listed in the column header and the testing sets are indicated in the row header of the table. Merged-zscore refers to the data set merged from the individual data set, each normalized separately by Z-score normalization. * indicates that the predictor was trained from all data sets except the testing set. NA stands for Not Available.
(0.04 MB PDF)

Figure S1 Prediction performance of the breast cancer gene signatures as a function of the number of genes. Gene nb refers to the number of genes.
(1.12 MB TIF)
