# Supplementary material for: Can Survival Prediction Be Improved By Merging Gene Expression Data Sets?
Source: PLoS One. 2009 Oct 23;4(10):e7431. doi: 10.1371/journal.pone.0007431 (PMC2761544; doi:10.1371/journal.pone.0007431)
Supplement: Table S1 — Cross-data set performance of breast cancer predictors trained on the individual and combined data sets (adjusted by ComBat) with respect to OS. Significant HR (p<0.05) are shown in bold. The training sets are listed in the column header and the testing sets are indicated in the row header of the table. * indicates that the predictor was trained from all data sets except the testing set. NA stands for Not Available. (0.04 MB PDF) [file pone.0007431.s001.pdf]

	GSE1456	GSE1992	GSE4335	Vijver	GSE3143	Merged-ComBat*
GSE1456	NA	2.01(0.95-4.26) p=0.06	2.50(1.16-5.37) p=**0.017**	6.55(2.5-17.19) p=**1e-04**	3.33(1.47-7.53) p=**0.003**	7.12(2.71-18.66) p=**6.5e-05**
GSE1992	3.06(1.22-7.66) p=**0.01**	NA	3.44(1.49-7.96) p=**0.0032**	4.82(1.81-12.81) p=**0.001**	5.08(1.9-13.57) p=**0.001**	4.22(1.69-10.54) p=**0.002**
GSE4335	1.78(0.86-3.66) p=0.11	1.59(0.7-3.62) p=0.26	NA	2.84(1.3-6.21) p=**p=0.008**	2.27(1.07-4.8) p=**0.03**	2.53(1.19-5.35) p=**0.015**
Vijver	5.96(3.14-11.32) p=**4.8e-08**	3.99(2.37-6.74) p=**2.02e-07**	3.38(2.05-5.57) p=**1.7e-06**	NA	3.43(2.07-5.68) p=**1.83e-06**	6.20(3.40-11.30) p=**2.5e-09**
GSE3143	$p \geq 0.05$	$p \geq 0.05$	$p \geq 0.05$	1.99(1.08-3.64) p=**0.02**	NA	2.13(1.17-3.87) p=**0.012**

Table S1: Cross-data set performance of breast cancer predictors trained on the individual and combined data sets (adjusted by ComBat) with respect to OS. Significant HR ($p < 0.05$) are shown in bold. The training sets are listed in the column header and the testing sets are indicated in the row header of the table. * indicates that the predictor was trained from all data sets except the testing set. NA stands for Not Available.
