# Supplementary material for: Can Survival Prediction Be Improved By Merging Gene Expression Data Sets?
Source: PLoS One. 2009 Oct 23;4(10):e7431. doi: 10.1371/journal.pone.0007431 (PMC2761544; doi:10.1371/journal.pone.0007431)
Supplement: Table S2 — Cross-data set performance of breast cancer predictors trained on the individual and combined data sets (normalized by Z-score normalization) with respect to OS. Significant HR (p<0.05) are shown in bold. The training sets are listed in the column header and the testing sets are indicated in the row header of the table. Merged-zscore refers to the merged data set combined from the individual data sets, each normalized separately by Z-score normalization. * indicates that the predictor was trained from all data sets except the testing set. NA stands for Not Available. (0.04 MB PDF) [file pone.0007431.s002.pdf]

| | GSE1456 | GSE1992 | GSE4335 | Vijver | GSE3143 | Merged-zscore* |
|---|---|---|---|---|---|---|
| GSE1456 | NA | 2.54(1.08-5.94) p=**0.032** | 2.63(1.22-5.65) p=**0.014** | 8.86(3.08-25.47) p=**5.1e-05** | 2.61(1.19-5.74) p=**0.017** | 4.74(2.02-11.12) p=**0.00033** |
| GSE1992 | p $\geq$ 0.05 | NA | 3.67(1.59-8.51) p=**0.0024** | 4.34(1.74-10.82) p=**0.0017** | 3.31(1.32-8.29) p=**0.011** | 4.28(1.71-10.69) p=**0.0018** |
| GSE4335 | 1.87(0.82-4.25) p=0.14 | 3.73(1.15-12.14) p=**0.029** | NA | 2.72(1.37-5.40) p=**0.0043** | 3.30(1.45-7.49) p=**0.0044** | 2.90(1.43-5.85) p=**0.003** |
| Vijver | 5.91(3.04-11.52) p=**1.8e-07** | 3.32(1.89-5.86) p=**3.3e-05** | 3.36(2.04-5.54) p=**1.9e-06** | NA | 3.61(2.10-6.21) p=**3.4e-06** | 5.21(2.90-9.33) p=**2.8e-08** |
| GSE3143 | p $\geq$ 0.05 | p $\geq$ 0.05 | p $\geq$ 0.05 | 2.10(1.16-3.82) p=**0.014** | NA | 1.93(1.06-3.49) p=**0.03** |

**Table S2: Cross-data set performance of breast cancer predictors trained on the individual and combined data sets (normalized by Z-score normalization) with respect to OS.** Significant HR ($p < 0.05$) are shown in bold. The training sets are listed in the column header and the testing sets are indicated in the row header of the table. Merged-zscore refers to the merged data set combined from the individual data sets, each normalized separately by Z-score normalization. * indicates that the predictor was trained from all data sets except the testing set. NA stands for Not Available.
