# Supplementary material for: Can Survival Prediction Be Improved By Merging Gene Expression Data Sets?
Source: PLoS One. 2009 Oct 23;4(10):e7431. doi: 10.1371/journal.pone.0007431 (PMC2761544; doi:10.1371/journal.pone.0007431)
Supplement: Table S3 — Cross-data set performance of breast cancer predictors trained on the individual and combined data sets (adjusted by ComBat) with respect to RFS. Significant AUC (>0.60) are shown in bold. The training sets are listed in the column header and the testing sets are indicated in the row header of the table. * indicates that the predictor was trained from all data sets except the testing set. NA stands for Not Available. (0.04 MB PDF) [file pone.0007431.s003.pdf]

	GSE1456	GSE1992	GSE4335	Vijver	GSE2990	GSE4922	GSE2034	Merged-ComBat*
GSE1456	NA	**0.69**	**0.67**	**0.76**	**0.77**	**0.72**	**0.72**	**0.73**
GSE1992	0.56	NA	**0.67**	**0.69**	**0.72**	**0.60**	0.56	**0.71**
GSE4335	**0.60**	**0.65**	NA	**0.68**	**0.61**	0.59	**0.65**	**0.64**
Vijver	**0.67**	**0.65**	**0.65**	NA	**0.63**	**0.64**	**0.66**	**0.67**
GSE2990	0.59	**0.67**	**0.61**	0.57	NA	0.53	0.59	**0.60**
GSE4922	**0.65**	0.57	0.56	**0.64**	**0.61**	NA	**0.64**	**0.64**
GSE2034	0.57	0.59	0.53	0.58	**0.60**	0.56	NA	0.58

Table S3: Cross-data set performance of breast cancer predictors trained on the individual and combined data sets (adjusted by ComBat) with respect to RFS. Significant AUC (> 0.60) are shown in bold. The training sets are listed in the column header and the testing sets are indicated in the row header of the table. * indicates that the predictor was trained from all data sets except the testing set. NA stands for Not Available.
