# Supplementary material for: Can Survival Prediction Be Improved By Merging Gene Expression Data Sets?
Source: PLoS One. 2009 Oct 23;4(10):e7431. doi: 10.1371/journal.pone.0007431 (PMC2761544; doi:10.1371/journal.pone.0007431)
Supplement: Table S4 — Cross-data set performance of breast cancer predictors trained on the individual and combined data sets (normalized by Z-score normalization) with respect to RFS. Significant AUC (>0.60) are shown in bold. The training sets are listed in the column header and the testing sets are indicated in the row header of the table. Merged-zscore refers to the data set merged from the individual data set, each normalized separately by Z-score normalization. * indicates that the predictor was trained from all data sets except the testing set. NA stands for Not Available. (0.04 MB PDF) [file pone.0007431.s004.pdf]

	GSE1456	GSE1992	GSE4335	Vijver	GSE2034	GSE2990	GSE4922	Merged-zscore*
GSE1456	NA	**0.70**	**0.67**	**0.76**	**0.71**	**0.78**	**0.73**	**0.74**
GSE1992	0.58	NA	**0.62**	**0.68**	0.56	**0.62**	0.59	0.59
GSE4335	**0.65**	**0.70**	NA	**0.68**	**0.65**	0.58	**0.65**	**0.61**
Vijver	**0.68**	**0.64**	**0.65**	NA	**0.66**	**0.68**	**0.67**	**0.69**
GSE2034	0.58	0.56	0.51	**0.60**	NA	**0.62**	0.58	0.59
GSE2990	**0.71**	**0.70**	**0.61**	**0.73**	**0.64**	NA	**0.67**	**0.72**
GSE4922	**0.65**	0.57	0.59	**0.66**	**0.63**	**0.65**	NA	**0.64**

Table S4: Cross-data set performance of breast cancer predictors trained on the individual and combined data sets (normalized by Z-score normalization) with respect to RFS. Significant AUC (> 0.60) are shown in bold. The training sets are listed in the column header and the testing sets are indicated in the row header of the table. Merged-zscore refers to the data set merged from the individual data set, each normalized separately by Z-score normalization. * indicates that the predictor was trained from all data sets except the testing set. NA stands for Not Available.
