# Supplementary material for: Can Survival Prediction Be Improved By Merging Gene Expression Data Sets?
Source: PLoS One. 2009 Oct 23;4(10):e7431. doi: 10.1371/journal.pone.0007431 (PMC2761544; doi:10.1371/journal.pone.0007431)
Supplement: Table S5 — HR of breast cancer predictors trained on the individual and combined data sets (adjusted by ComBat) with respect to RFS. Significant HR (p<0.05) are shown in bold. The training sets are listed in the column header and the testing sets are indicated in the row header of the table. * indicates that the predictor was trained from all data sets except the testing set. NA stands for Not Available. (0.04 MB PDF) [file pone.0007431.s005.pdf]

	GSE1456	GSE1992	GSE4335	Vijver	GSE2034	GSE2990	GSE4922	Merged-ComBat*
GSE1456	NA	3.11(1.58-6.12) p=**0.001**	2.38(1.27-4.44) p=**0.0063**	7.82(3.28-18.67) p=**p=3.60e-06**	3.73(1.89-7.34) p=**0.0001**	6.88(2.69-17.59) p=**5.56e-05**	4.60(2.11-9.99) p=**0.0001**	6.19(2.85-13.45) p=**4.22e-06**
GSE1992	2.30(1.12-4.71) p=**0.022**	NA	2.63(1.31-5.25) p=**0.0061**	2.58(1.24-5.39) p=**0.011**	3.02(1.47-6.19) p=**0.0025**	2.82(1.23-6.47) p=**0.014**	2.20(1.05-4.59) p=**0.035**	2.90(1.41-5.94) p=**0.0035**
GSE4335	$p \geq 0.05$	$p \geq 0.05$	NA	$p \geq 0.05$	$p \geq 0.05$	$p \geq 0.05$	$p \geq 0.05$	$p \geq 0.05$
Vijver	3.94(2.5-6.21) p=**3.45e-09**	2.94(1.95-4.42) p=**2.65e-07**	2.22(1.51-3.28) p=**5.60e-05**	NA	2.68(1.79-4.01) p=**1.83e-06**	3.6(2.27-5.72) p=**5.45e-08**	2.48(1.66-3.7) p=**9.40e-06**	3.08(2.05-4.61) p=**5.25e-08**
GSE2034	1.83(1.23-2.74) p=**0.0027**	1.86(1.26-2.73) p=**0.0015**	$p\geq 0.05$	1.80(1.22-2.65) p=**0.003**	NA	1.80(1.22-2.65) p=**0.0029**	1.85(1.23-2.77) p=**0.0037**	1.83(1.24-2.69) p=**0.002**
GSE2990	$p \geq 0.05$	$p \geq 0.05$	$p \geq 0.05$	$p \geq 0.05$	$p \geq 0.05$	NA	$p\geq 0.05$	$p \geq 0.05$
GSE4922	2.50(1.60-3.89) p=**4.7e-05**	$p \geq 0.05$	1.72(1.13-2.61) p=**0.011**	2.15(1.41-3.27) p=**0.0003**	1.99(1.31-3.04) p=**0.0013**	2.54(1.62-3.96) p=**4.3e-05**	NA	2.36(1.55-3.61) p=**7.1e-05**

Table S5: HR of breast cancer predictors trained on the individual and combined data sets (adjusted by ComBat) with respect to RFS. Significant HR ($p < 0.05$) are shown in bold. The training sets are listed in the column header and the testing sets are indicated in the row header of the table. * indicates that the predictor was trained from all data sets except the testing set. NA stands for Not Available.
