# Supplementary material for: Can Survival Prediction Be Improved By Merging Gene Expression Data Sets?
Source: PLoS One. 2009 Oct 23;4(10):e7431. doi: 10.1371/journal.pone.0007431 (PMC2761544; doi:10.1371/journal.pone.0007431)
Supplement: Table S6 — HR of breast cancer predictors trained on the individual and combined data sets (normalized by Z-score normalization) with respect to RFS. Significant HR (p<0.05) are shown in bold. The training sets are listed in the column header and the testing sets are indicated in the row header of the table. Merged-zscore refers to the data set merged from the individual data set, each normalized separately by Z-score normalization. * indicates that the predictor was trained from all data sets except the testing set. NA stands for Not Available. (0.04 MB PDF) [file pone.0007431.s006.pdf]

	GSE1456	GSE1992	GSE4335	Vijver	GSE2034	GSE2990	GSE4922	Merged-zscore*
GSE1456	NA	3.51(1.62-7.63) p=**0.0015**	3.16(1.65-6.06) p=**0.00053**	6.25(2.88-13.59) p=**1.05e-05**	2.90(1.50-5.63) p=**0.0016**	6.18(2.73-14) p=**1.2e-05**	5.25(2.32-11.89) p=**7e-05**	6.51(2.87-14.75) p=**7e-06**
GSE1992	$p \geq 0.05$	NA	2.96(1.46-5.98) p=**0.0026**	2.72(1.33-5.55) p=**0.006**	2.06(1.04-4.07) p=**0.037**	2.02(1.00-4.08) p=0.05	2.35(1.13-4.90) p=**0.022**	2.12(1.06-4.22) p=**0.032**
GSE4335	$p \geq 0.05$	4.77(1.14-19.86) p=**0.032**	NA	$p \geq 0.05$	$p \geq 0.05$	$p \geq 0.05$	$p \geq 0.05$	$p \geq 0.05$
Vijver	3.37(2.17-5.21) p=**5.05e-08**	3.40(2.17-5.32) p=**8.60e-08**	2.37(1.60-3.49) p=**1.42e-05**	NA	2.57(1.73-3.83) p=**3.45e-06**	3.19(2.08-4.88) p=**1.0e-07**	3.07(2.03-4.64) p=**1.11e-07**	3.18(2.10-4.82) p=**4.3e-08**
GSE2034	1.83(1.23-2.72) p=**0.0029**	1.80(1.22-2.66) p=**0.0032**	$p \geq 0.05$	1.95(1.32-2.87) p=**0.0001**	NA	1.77(1.20-2.62) p=**0.0043**	1.60(1.08-2.35) p=**0.018**	1.76(1.19-2.61) p=**0.0041**
GSE2990	3.22(1.39-7.45) p=**0.0064**	2.54(1.22-5.28) p=**0.012**	$p \geq 0.05$	3.39(1.57-7.33) p=**0.001**	3.14(1.48-6.68) p=**0.003**	NA	2.31(1.09-4.87) p=**0.029**	3.79(1.70-8.46) p=**0.0011**
GSE4922	2.25(1.45-3.49) p=**0.00028**	$p \geq 0.05$	1.83(1.20-2.78) p=**0.0049**	2.33(1.53-3.56) p=**8.76e-05**	2.01(1.31-3.06) p=**0.0012**	2.56(1.67-3.94) p=**1.8e-05**	NA	2.25(1.47-3.43) p=**0.00017**

Table S6: HR of breast cancer predictors trained on the individual and combined data sets (normalized by Z-score normalization) with respect to RFS. Significant HR ($p < 0.05$) are shown in bold. The training sets are listed in the column header and the testing sets are indicated in the row header of the table. Merged-zscore refers to the data set merged from the individual data set, each normalized separately by Z-score normalization.* indicates that the predictor was trained from all data sets except the testing set. NA stands for Not Available.
