# Supplementary figures and images for: Can Survival Prediction Be Improved By Merging Gene Expression Data Sets?
Source: PLoS One. 2009 Oct 23;4(10):e7431. doi: 10.1371/journal.pone.0007431 (PMC2761544; doi:10.1371/journal.pone.0007431)

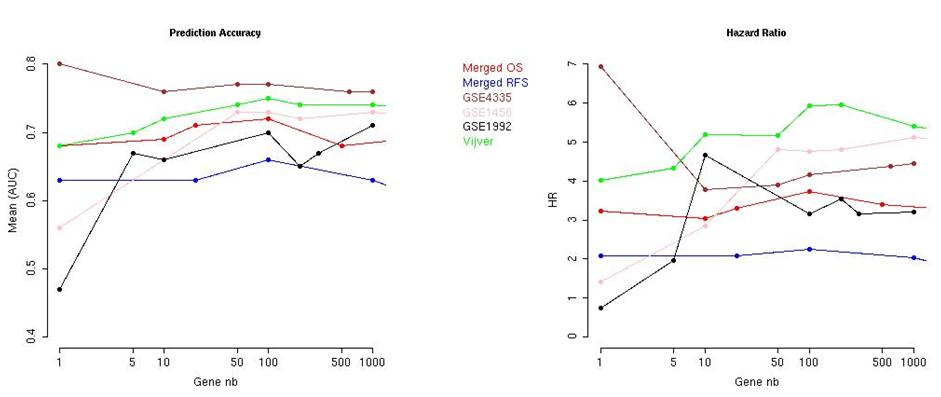

Supplement: Figure S1 — Prediction performance of the breast cancer gene signatures as a function of the number of genes. Gene nb refers to the number of genes. (1.12 MB TIF) [file pone.0007431.s007.tif]
